# Cognitive Profile of ADHD in Older Adults: A Systematic Review

**DOI:** 10.1177/10870547251385758

**Published:** 2025-10-27

**Authors:** Natividad Pardo-Palenzuela, Iban Onandia-Hinchado, Unai Diaz-Orueta

**Affiliations:** 1 Universidad de Almería, Spain; 2 Clínica Neurodem, Almería, Spain; 3 Psicología Amorebieta, Spain; 4 Universidad Internacional de La Rioja (UNIR), Spain; 5 Maynooth University, Ireland

**Keywords:** neuropsychological assessment, adult ADHD, cognitive domains, aging, older adults

## Abstract

**Objectives:**

ADHD is now recognized as a common condition in adulthood, but the evidence supporting a separate characterization of a cognitive profile for ADHD in older adults is scarce. Consequently, the goal of the current study was to conduct a systematic review that helps clarify the cognitive characteristics of ADHD in older individuals.

**Method:**

We conducted a systematic review with narrative synthesis, considering studies on older adults with ADHD and research on cognitive domains involved in adults 50 years old and older with a confirmed diagnosis of ADHD, in three electronic databases (PubMed, Web of Science, and Embase). Ten studies (3 longitudinal and 7 cross-sectional) with clearly separated cognitive data for older adults with ADHD were included in this review.

**Results:**

Results showed an overall worse performance in attention and episodic memory for older adults with ADHD compared to their younger counterparts and older healthy controls. Evidence concerning executive functions was mixed, with some studies showing a worse performance in working memory compared to older healthy controls, but with other studies showing a similar or even better performance than younger adults with ADHD.

**Conclusions:**

A cognitive characterization of ADHD in older adults requires further research to clarify whether it can be considered a separate entity and how to establish a differential diagnosis with other age-related conditions. Moreover, there is a need for internationally agreed common neuropsychological assessment protocols that set boundaries between younger and older adults with ADHD.

## Introduction

ADHD has been reported as the most frequent neurodevelopmental disorder diagnosed during childhood, but it may extend over the lifespan. Diverse studies (Aboitiz et al., 2014; Ginsberg et al., 2014) remark on its presence as a common condition in adults, with an estimated prevalence of 2.5% to 5% of the general population, and with later studies recognizing it as a neurodevelopmental syndrome that may impact differently depending on how these prevalence data were collected. Subsequently, prevalence in adults older than 50 years ranges from 2% (Thorell et al., 2017) and 2.8% (Michielsen et al., 2012) to 3.3% (Goodman et al., 2016; Torgersen et al., 2016). On a further step, Dobrosavljevic et al. (2020) stated that validated scales have allowed identifying elevated levels of ADHD symptoms in a large number of older adults, and that less than half of clinically diagnosed older adults with ADHD are actually treated. In their meta-analysis, these authors compiled 20 relevant studies with 32 datasets, comprising 41,420 individuals with ADHD. Depending on the assessment methods, the differences in pooled prevalence estimates were significant: 2.18% (95% CI [1.51, 3.16]) based on research diagnosis via validated scales, 0.23% [0.12, 0.43] based on clinical ADHD diagnosis, and 0.09% [0.06, 0.15] based on ADHD treatment rates.

The absence of age-specific diagnostic criteria for adult ADHD may also explain, to some extent, the low numbers in the prevalence of ADHD in older adults. Sharma et al. (2020) state that the age-of-onset criterion should be discarded when performing an ADHD diagnosis in adults and older adults for the first time in their lives. Moreover, these authors report evidence to suggest that some high-functioning individuals with ADHD can maintain function through different cognitive or behavioral strategies or due to high IQ, which may mask ADHD symptoms or at least offer compensation for deficits in other processes. In addition, a relevant number of children who meet ADHD criteria may not be diagnosed due to reduced access to healthcare resources, particularly in the context of families with lower socioeconomic status. In addition, as Kooij et al. (2016) state, trying to base the diagnosis on an age-of-onset criterion may not be appropriate for an ADHD diagnosis in late life, due to problems especially linked to long-term memory function in old age, since findings will not represent the majority of older adults with ADHD symptoms (i.e., with many unable to provide reliable evidence of childhood symptoms and/or many who maintained high levels of functioning in earlier life due to environmental or social support systems). Subsequently, the fallibility of memory and self-knowledge in ADHD, in addition to the expected age-related decline in memory functioning, may lead to the conclusion that older adults with ADHD are particularly unreliable storytellers when it comes to providing valid narratives about the onset of symptoms in their childhood.

In terms of treatment strategies, in a recent review, Dobrosavljevic et al. (2023) stated that international treatment guidelines provide little or no recommendations for older adults with ADHD. These authors suggest a focus on a multimodal and multidisciplinary approach for older adults, considering general physical health, comorbidities, and other medications before and during pharmacological interventions, due to risks associated with high physical comorbidity rates and increased cardiovascular risk in older adults. Hence, there is a need to identify what constitutes ADHD in older age in order to display adequate treatment strategies.

A primary challenge for characterizing ADHD in older adults is the lack of knowledge about the condition at this late life stage. Fischer et al. (2012) showed that much of the data that could support ADHD diagnosis in older adults may be inaccessible (e.g., old school records). Ginsberg et al. (2014) reported that it is still difficult to find professionals able to diagnose adult ADHD across most European countries. In addition, as Fischer et al. (2012) state, behaviors representative of ADHD may have been better tolerated, and therefore probably overlooked in previous generations. More specifically, Ginapp et al. (2022) stated that assessing and diagnosing ADHD in adults was considered difficult, and reasons for these difficulties included previous misdiagnoses, lack of psychiatric resources, and physicians’ prejudices in relation to adult ADHD. However, according to Kooij et al. (2016), there seems to be increased reported symptomatology by individuals in their 60s compared to older groups between 71 and 94 years old, although the specific ways in which ADHD manifests in late life, and which difficulties older adults with ADHD must deal with, are still unclear. It seems established that, while hyperactivity/impulsivity tends to decline in adulthood, inattention continues largely in a more stable manner. Similarly, the role of interpersonal relationship problems and mood disorders (especially anxiety and depressive symptoms), eating disorders, sleep disturbances, substance use, and other comorbidities widely reported in ADHD must also be explored in older adults.

A closer look at the specific symptoms of ADHD in older adults shows that much of the existing research has targeted quality-of-life aspects of ADHD at this age. For example, Brod et al. (2012) found that quality of life is impacted by the continuous negative effect of ADHD symptoms over the years and cumulative impairments on individuals’ professional, economic, social, and emotional well-being, Similarly, Semeijn et al. (2013) and Kooij et al., (2016) linked ADHD in older adults with chronic physical illnesses (such as chronic nonspecific lung diseases or cardiovascular diseases) and poorer self-perceived health, but not with lifestyle variables. However, as Brod et al. (2012) state, symptoms do not seem to be different from those in younger adults. Semeijn et al. (2016) found comparable results based on data from the Longitudinal Aging Study of Amsterdam, stating that diagnostic criteria developed for younger adults may be used for older adults, although they recognized the limitation of using retrospective, self-reported data. Philipp-Wiegmann et al. (2016), also retrospectively, found that ADHD subjects reported negative impacts due to ADHD-associated behavior over the life span, with impairments in family life, social relationships, money management, and daily life organization, mainly caused by low self-confidence, being quick-tempered, and due to defiantness. More recently, Thorell et al. (2022) reported that older people with ADHD exhibited a low level of self-esteem, quality of life, and social connection, as well as a high degree of poor psychiatric and somatic health. Later, Nagamine (2023) reported that while older ADHD patients show lower hyperactivity symptomatology than younger adults, they show higher social impairment and chronic pain, with the gut microbiota as an important therapeutic target that requires further research.

The distinction between ADHD symptomatology and other health conditions in older adults makes the differential diagnosis in late life more complex and difficult. ADHD symptoms like frontal-executive impairments, inattentiveness, forgetfulness, or challenges involving activities of daily living or socialization, among others, can appear to be confounded with other conditions such as Mild Cognitive Impairment (MCI) or dementia (Ivanchak et al., 2012; Ngo, 2023, Surman & Goodman, 2017; Torgersen et al.; 2016), which are concurrent with an increase of medication intake, sleep disorders, pain, as well as with visual and auditory impairments. Similarly, as Callahan et al. (2017) state, current diagnostic criteria for MCI or dementia may not be valid in individuals with a previous, premorbid history of attentional and/or hyperactive difficulties. Moreover, symptomatology like emotional dysregulation, agitation, irritability, or distractibility overlaps with other psychiatric disorders such as depression, anxiety, substance use disorders, personality disorders, or bipolar disorders.

Some recent studies have attempted both to clarify the association between ADHD and neurodegenerative conditions linked to old age, as well as specific symptomatology associated with ADHD in late life. Golimstok et al. (2011) identified a significantly higher risk of Dementia with Lewy Bodies amongst patients with past ADHD symptoms. More recently, Sasaki et al. (2022) conducted an observational study on clinical features of ADHD in older adults in a dementia clinic, and found that 1.6% of patients who were initially suspected of having dementia were actually diagnosed with ADHD, and that the “late-onset” described in previous studies should rather be considered as a “late-manifestation.” One year later, Levine et al. (2023) found that the presence of adult ADHD was significantly associated with an increased dementia risk, and Becker et al. (2023) stated that ADHD is differentially associated with all-cause and subtypes of dementia, and that some people with ADHD may have been mislabeled as controls in selected studies. Only one recent systematic review by Prentice et al. (2021) aimed to compare neuropsychological performance in older adults with ADHD and older adults with Dementia with Lewy Bodies (DLB). These results revealed isolated working memory deficits for late-life ADHD, versus more extended performance deficits in areas of attention, memory, language, and visuoperceptual abilities for DLB. However, results interpretation was limited and affected by small sample sizes and a lack of data for some cognitive domains. Only more recently, Golimstok et al. (2024) found increased evidence that ADHD is independently associated with an increased risk of Lewy Bodies disease, dementia, and non-amnestic MCI.

Finally, to add more challenges to a proper diagnosis and subsequent treatment of ADHD in older adults, there are very few studies reporting on specific cognitive functions affected in older adults with ADHD. Kooij et al. (2016) stated that attention/working memory was negatively associated with ADHD in older adults, although this association was mediated by depressive symptoms, and once these symptoms were controlled, cognitive problems disappeared. Luders et al. (2016) concluded that the corpus callosum has a thickness process (mediated by depression symptoms), and that this process and its final result could also describe inattentive and hyperactivity features. In a review by Surman and Goodman (2017), the attempt to characterize ADHD in older adults from a neuropsychological point of view was not found to represent a definitive presence of the disorder. For these authors, diverse performance in neuropsychological tests in ADHD studies does not help confirm the presence of ADHD, so these tests should be used as a resource to qualify and quantify cognitive deficits, but not for diagnostic confirmation.

To our knowledge, the only recent scoping review considering cognitive features of ADHD in older adults was performed by Fischer and Nilsen (2024), who intended to present an overview on the research on symptoms, comorbidities, and associated challenges among older adults with ADHD, with results pointing specifically to depression and anxiety as the main comorbidities, and difficulty with relationships and social isolation as the main challenges. In terms of cognition, they seemed to limit the problems to attention and working memory, but without a clear delimitation of cognitive deterioration profiles in older adults with ADHD. However, such profiles should confirm whether it is possible to identify specific differences with younger adults, as well as specific differential cognitive features from other conditions associated with old age, such as Alzheimer’s disease and other dementias.

Subsequently, taking into account all the previous considerations and challenges, the goal of the current study is to conduct a systematic review of the literature to identify cognitive domains specifically associated with ADHD in older adults, in order to confirm the existence of a differentiated cognitive profile for this age population.

## Methods

### Searching Strategy

The method of this systematic review was developed in accordance with the Preferred Reporting Items for Systematic Reviews and Meta-Analysis (PRISMA) criteria (Page et al., 2021). Scientific studies were identified by searching electronic databases (PubMed, Web Of Science, Embase) using the following syntax adapted to the searching requirements in each database: (“adult ADHD” or “adult attention hyperactivity disorder”) AND (“neuropsychology” or “cognition” or “executive functions” or “attention” or “memory” or “language” or “visuospatial” or “visuoconstructive” or “social cognition” or “dysexecutive syndrome” or “impulsivity” or “inhibition” or “motor” or “reasoning”) AND (“elderly” OR “older adults”). The period was set to identify research developed in the last 20 years, ranging from 1st January 2005 to 31st December 2024. No specific exclusion terms were added in order to extend the scope of the review as much as possible.

### Selection Strategy, Screening, and Extraction

Combination of electronic databases with the examination of grey literature raised a total of 493 references, which were subsequently filtered in order to remove 72 duplicates. That led to a total of 421 studies to be screened as potential to be included in the results. At this point, only studies in English and Spanish were included and an exhaustive exclusion strategy was followed in order to narrow the scope into the study goals, thus leading to the exclusion of the following studies. The exclusion criteria and the quality review strategy used are the same ones that we used in a previous systematic review (Onandia-Hinchado et al., 2021). These criteria are listed below:

- All studies in a language other than English or Spanish.
- Genetic studies on ADHD.
- Studies with a primary focus on fMRI, EEG, and ERP.
- Studies with mixed samples of children, adolescents, adults, and older with ADHD that did not provide separate data for older adults with ADHD.
- Studies focused on ADHD with comorbidities, when the main focus of the study was those comorbidities, or when cognitive performance outcomes attributable to ADHD were not distinguishable from those attributed to the comorbidities. This may include alcohol or other drug consumption, other psychiatric disorders (e.g., schizophrenia, bipolar disorder, eating disorders, gambling, internet addiction, etc.), and personality disorders (mainly, antisocial, or borderline personality disorders).
- Studies focused on the effects of specific interventions, both pharmacological and non-pharmacological.
- Editorials, letters to editors, errata, corrigenda, and book reviews.

Each of the authors reviewed a total of two-thirds of the selected articles (281) so each study was screened independently by at least two out of three researchers in order to avoid selection biases, and the conflicts were solved by all the three research team members. This selection and exclusion strategy led to the exclusion of another 398 studies, leading to a final selection of 23 articles to be assessed for quality purposes.

### Quality Review Strategy

The quality review of the 23 selected articles led to the exclusion of 13 studies. This quality review was not based on standardized tools, but instead, on specific dichotomic features that were either present or absent in the reviewed papers. The goal was to ensure that articles (1) included samples of older adults with a confirmed diagnosis of ADHD, with no other psychiatric or cognitive comorbidities; (2) that included measures of cognitive domains; and (3) that had control groups with whom cognitive domains in the older adult ADHD samples could be compared. The establishment of these clear dichotomic yes/no criteria led to the exclusion of:

- Studies that focused on samples with ADHD symptoms, without a confirmed diagnosis.
- Studies that claimed focusing on adult ADHD, that had either no older adults or had mixed-aged samples with no separate data for older adults with ADHD.
- Studies that claimed focusing on cognitive domains, but which did not report cognitive measures (i.e., saccades, gait, motor aspects, driving abilities, etc.).
- Studies with no control group to which to compare results on different cognitive domains.
- Studies with psychiatric populations showing complex profiles with comorbidities.
- Studies with older adult ADHD mixed samples of individuals with and without comorbidities, and reported all results together, without separate data for older adults with ADHD.
- Studies with older adult ADHD samples with a primary focus on other non-cognitive variables.

This quality review strategy led to a final number of 10 studies (3 longitudinal and 7 cross-sectional studies) finally included in the systematic review.

## Results

**Figure 1.**
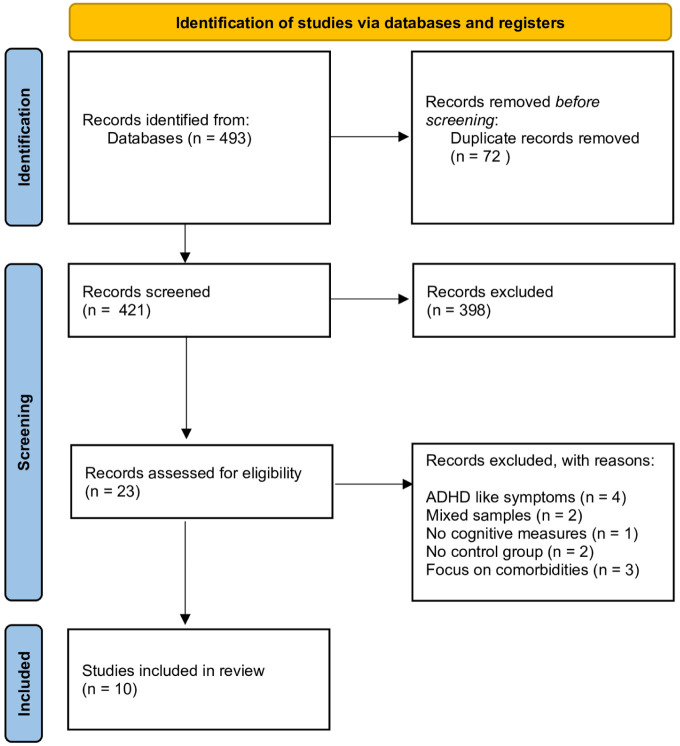
PRISMA diagram for the current systematic review. *Source*. Page MJ, et al. BMJ 2021;372:n71. doi: 10.1136/bmj.n71. This work is licensed under CC BY 4.0. To view a copy of this license, visit https://creativecommons.org/licenses/by/4.0/.

Figure 1 shows the PRISMA Diagram with the search and filtering process followed from the initial identification of potentially eligible studies to the final selection of 10 studies included in this systematic review. Table 1 shows the summaries of the ten studies finally included in the review.

**Table 1.** Studies Included in the Systematic Review.

Authors and publication year	Study design	Participants	Age	Domains assessed	Measures used	Summary of main results
Bijlenga et al. (2019)	Cross-sectional	*n* = 209 with ADHD (97) without ADHD (112)	55–79	- Impulsivity - Inattention	- The Quantitative Behavioral Test	Older age was associated with more inattention.
Callahan & Plamondon (2019)	Cross-sectional	*n* = 878 aged 18–59 (645) aged 60–85 (233)	18–85	- Working memory - Vigilance - Cognitive flexibility - Attention networks	- Digit span (WAIS-R) - Letter N-Back (CNB) - Test–Number/Letter (CNB) - The Penn Conditional Exclusion Test - Attention Network Test	The factor structure of ADHD symptomatology is consistent across adulthood and is associated with aspects of executive functioning. In older adults, ADHD symptoms predicted poorer working memory. There are no robust associations between ADHD symptomatology and executive functions.
Callahan et al. (2021)	Longitudinal	*n* = 1092	18–85	- Switching - Working Memory—Reaction Time - Processing Speed	- University of Pennsylvania Computerized Neurocognitive Battery (CNB) -Delis-Kaplan Executive Functioning System (D-KEFS) -Wechsler Memory Scale-Revised (WMS-R)	Inspection of associations between ADHD symptoms and EF revealed that there were no significant associations between current ADHD symptoms and any variable.
Callahan et al. (2022)	Cross-sectional	*n* = 106 ADHD (40) MCI (29) controls (37)	50–85	- Attention - Episodic Memory - Language - Executive Abilities	- Forward digit span, Trails A, digit-symbol coding, Stroop word-reading, and color-naming - Logical Memory Short Story - California Verbal Learning Test (CVLT), Rey Osterrieth Complex Figure (ROCF) - Boston Naming Test (BNT), phonemic, and semantic fluency - Wisconsin Card Sorting Test (WCST), backward digit span, Stroop interference	Memory was impaired with an encoding deficit in ADHD (supported by frontal lobe thinning). This group displayed normal executive functioning.
Callahan et al. (2024)	Cross-sectional	*n* = 76 ADHD (39) controls (37)	48–81	- Verbal Memory - Visual Memory - Processing Speed - Language - Visuoconstruction - Executive Functions	- Short- and long-term verbal memory (forward digit span, California Verbal Learning Test, Logical Memory) - Visual memory (Visual Recognition, Rey Complex Figure) - Processing speed (coding, trails A, Stroop word-reading, and color-naming) - Language (Boston Naming Test, semantic fluency) - Visuoconstruction (clock drawing, Rey Complex Figure copy) - Executive function (backward digit span, Trails B, phonemic fluency, Stroop inhibition, Wisconsin Card Sorting Test).	ADHD participants had lower composite scores than controls on processing speed and executive functioning. Results showed a moderated effect of ADHD on cognition by White Matter Hyperintensities (WMH). ADHD was negatively associated with memory, speed, and executive functioning at high and medium (but not low) levels of WMH, despite overall lower vascular burden in ADHD.
Das et al. (2014)	Longitudinal	*n* = 4155 middle-age 48–52 (2,182) older-age 68–74 (1,973)	48–52 68–72	- Language - Attention - Executive Functions - Memory - Working Memory - Processing Speed	- Spot-the-Word Test (SWT), TMT, Symbol-Digit Modalities Test (SDMT), CVLT, Digit span backward (DSB), Simple and Choice Reaction Time (SRT and CRT).	Although ADHD symptoms persist, they are less common in older adults compared to middle-aged adults.
Mendonca et al. (2021)	Cross-sectional	*n* = 107 controls (41) MCI (40) ADHD (26)	60–76	- Semantic Memory—Executive Function - Visuospatial Abilities - Visual Tracking - Cognitive Flexibility - Attention - Working memory	- Mini Mental State Examination (MMSE) - Rey Auditory Verbal Learning Test (AVLT) - Measure of verbal episodic memory Block Design Test -Trail-making Test (TMT) -Digit Span Test - Verbal fluency test (Semantic and phonemic)	Participants with ADHD showed poorer performance than controls in episodic memory and executive function, with large effect sizes. Verbal memory impairments in ADHD were identified in acquisition, delayed recall, and recognition processes compared to controls.
Semeijn et al (2015)	Longitudinal Aging Study Amsterdam (LASA).	*n* = 231	60–94	- Executive—Functions - Information processing speed - Memory - Attention Working Memory	- MMSE - Raven’s Colored Progressive Matrices - Auditory Verbal Learning Test - Alphabet Coding Task-15 (ACT-15) - Stroop Color-Word Test - Trail Making Task - Word Fluency Test - Digit Span.	ADHD diagnosis and ADHD severity were only negatively associated with cognitive functioning in the attention/working memory domain.
Thorell et al. (2017)	Cross-sectional	*n* = 156 older adults with ADHD (42) healthy controls (58) younger adults ADHD (56)	18–75	- Executive Functions - Working memory - Inhibition - Switching	- D-KEFS - WAIS-IV - Adult Executive Function Inventory (ADEXI)	Older adults with ADHD differed from controls with regard to working memory, inhibition, switching, and delay-related behaviors. In comparison to younger adults with ADHD, they performed at a similar level in relation to working memory and planning, but significantly better with regard to inhibition, switching, fluency, speed of processing, and delay aversion. Despite several significant group differences relative to controls, person-oriented analyses demonstrated that a majority of older adults with ADHD performed within the average range on each test and 20% showed no clear deficit within any neuropsychological domain.
Thorell et al. (2019)	Cross-sectional	*n* = 156 older adults with ADHD (42) healthy controls (58) younger adults ADHD (56)	60–75	- Executive Functions - Working memory - Inhibition - Switching	- D-KEFS - WAIS-IV - ADEXI	ADHD symptom levels and executive functioning deficits explained about 40% of the variance in Life Productivity and Relationships among older adults with ADHD and about 20% in Life Outlook and Psychological Health.

In terms of general functioning, results are consistent with heterogeneity models identifying different neuropsychological subtypes in ADHD and a subgroup of patients without any clear neuropsychological deficits (Thorell et al. 2017). However, studies report that ADHD symptoms either manifest similarly across adulthood (Callahan & Plamondon, 2019), that older adults with ADHD do not show any clear deficit in any neuropsychological domain (Thorell et al., 2017) and that they may even improve in older compared to middle-aged adults with ADHD, as shown in the longitudinal study led by Das et al. (2014).

In terms of individual cognitive domains, ADHD diagnosis and symptom severity in older adults was associated mainly with the attentional domain (i.e., the more the inattention, the more the symptom severity) as reported both by Bijlenga et al. (2019) in their cross-sectional study and previously by Semeijn et al. (2015) in their longitudinal research.

In relation to memory, Callahan et al. (2022) showed a memory encoding deficit in older adults with ADHD, supported by a frontal lobe thinning, and in a posterior research, Callahan et al. (2024) showed the role of vascular burden (i.e., white matter hyperintensities) as a moderating factor affecting memory performance in older adults with ADHD, despite an overall low level of vascular pathology in their sample. In addition, Mendonca et al. (2021) reported that older participants with ADHD showed poorer performances than controls in episodic memory with large effect-sizes, with verbal memory impairments identified for acquisition, delayed recall, and recognition processes in relation to controls.

For executive functions, results differ depending on the type of study reported, with some inconsistencies shown within cross-sectional and longitudinal studies reviewed, especially in relation to the working memory domain.

In cross-sectional studies, working memory was the main cognitive function associated with deficits in older adult ADHD individuals, but with mixed results, and with inconsistent findings for the rest of the executive functions. More specifically, Callahan and Plamondon (2019) reported poorer working memory in older adults. One additional study, comparing older adults with ADHD and controls, showed worse performance in the patient group with regard to not only working memory, but also for inhibition, switching, and delay-related behaviors (Thorell et al. 2019). Mendonca et al. (2021) reported comparable results for executive functions, and Callahan et al. (2022) provided additional findings on lower processing speed in older adults with ADHD compared to controls. However, both Callahan and Plamondon (2019) and Thorell et al. (2017) show, respectively, no association or even a trend of better executive performance in older adults. More specifically, Callahan and Plamondon (2019; excluding working memory, as stated above) showed no further clear or robust associations of older adult ADHD with other executive function deficits; and Thorell et al. (2017), who compared older to younger adults with ADHD, not only showed similar working memory and planning levels, but even significantly better performance in older versus younger adults with ADHD in relation to inhibition, switching, fluency, speed of processing, and delayed aversion.

Separately, in longitudinal studies reviewed, Das et al. (2014) showed that ADHD symptom severity declined in older adults, and Callahan et al. (2021) showed no clear or robust associations between ADHD symptoms in older adults and executive function deficits. Only Semeijn et al. (2015) reported longitudinally an association of working memory deficits with ADHD diagnosis and symptom severity.

## Discussion

This review highlights the key features that distinguish ADHD in older adults from healthy controls as well as from other conditions. The original goal was to use a method based on the identification of individual cognitive functions or domains to better characterize the cognitive profile of ADHD in older adults. Unfortunately, the confusion around the diagnosis of ADHD in older age and the mixed outcomes in relation to how ADHD evolves during adult life have led to a difficult definition of the condition in late stages of life. However, despite the limitations of available studies, it seems possible to offer some insight into the traditional profile comprising attention, memory, and executive functions domains, together with some considerations about processing speed.

Overall, the heterogeneity of profiles observed in older adults seems to match the one seen in younger adults with ADHD (Callahan & Plamondon, 2019; Thorell et al., 2017), but some studies report that general ADHD symptomatology declines with age (Das et al., 2014). A closer look at individual cognitive domains reported a decline in attention (Bijlenga et al., 2019; Semeijn et al., 2015), episodic memory (including encoding or acquisition; Callahan et al., 2022, Mendonca et al., 2021; as well as delayed recall and recognition; Mendonca et al., 2021), and working memory (Callahan & Plamondon, 2019; Semeijn et al., 2015), while the usually recognizable profile of decline in executive functions was not found in older adults with ADHD (Callahan et al., 2021; 2022; Callahan & Plamondon, 2019) and they even seemed to improve in relation to speed of processing, inhibition, switching, fluency, and delayed aversion compared to younger counterparts (Thorell et al., 2017).

Limitations of this systematic review are quite obvious. The main goal of defining a differential profile in older adults with ADHD in comparison to healthy controls but also in comparison to younger adults with ADHD led to a confounding profile, where older adults with ADHD seemed to perform worse than control older adults, but not consistently worse than young adults with ADHD. The reasons for this apparent improvement in cognitive aspects related to executive function, processing speed and inhibition compared to younger adults require further research.

Additionally, to check the possibilities to focus on an ADHD-only, domain based, cognitive profile, the role of comorbidities has not been considered in this review. In terms of findings, the main limitation is the lack of studies clearly characterizing cognition in older adults with ADHD, which demands a clearer rationale for distinguishing between ADHD in older adults as a differentiated entity versus adult ADHD individuals who get older. The additional difficulties to distinguish whether cognitive symptoms in older adults belong to ADHD or to another type of dementia leading MCI (Ngo, 2023) complicates the picture further.

On the basis of this systematic review, we believe that future studies with older adults with ADHD need to provide further clarity to differential diagnosis with other age-associated conditions (i.e., neurodegenerative diseases) and develop a more precise cognitive and functional profile of the condition. Reviewed research on the area is minimal, and discrepancies in terms of sample sizes and study methodologies make it hard to establish clear diagnostic conclusions. As we stated before, if regular adult ADHD diagnosis already comprises mixed approaches in terms of diagnostic criteria and approaches, the picture is more blurred when it comes to older adults with ADHD. Comorbidities were left out of this review in the aim to establish a cognitive profile for ADHD in older adults, but their role in the symptomatology of ADHD cannot be ignored as it may limit the generalization of the results described here. Subsequently, it is essential to consider and establish their impact on older adult ADHD in future research and clinical work. It would be desirable to overcome the heterogeneity in neuropsychological assessment protocols and approaches, to develop common international neuropsychological assessment protocols that comprise domains described in this review, as well as others probably not clearly captured in the studies up to date.

Concerning evaluation, there are specific instruments for the adult population that assist in the diagnosis of ADHD through the identification of symptoms, such as the Adult ADHD Self-Reported Scale (ASRS; Kessler et al., 2005) or the Wender Utah Rating Scale (WURS; Ward et al., 1993). Additionally, directed clinical interviews have been developed that help develop a more general approach based on the main symptoms according to DSM V, with common examples that reference both childhood and the current moment, covering all diagnostic criteria, such as the Diagnostic Interview for ADHD in Adults (DIVA-5; Kooij & Francken, 2010), which is highly recommended for diagnostic use. At a neuropsychological assessment level, we do not find ADHD specific instruments for the measurement of cognitive performance in adult ADHD. Instead, both in clinical practice and research, tests and subtests from the general adult population that have age-appropriate validations are used, such as the Stroop Color and Word Test (Golden et al., 2002), The Trail Making Test (Reitan & Wolfson, 1985), or the Delis-Kaplan Executive Function System (D-KEFS; Delis et al., 2001), among others, and some subtests from the Wechsler Adult Intelligence Scale, currently in its fifth edition (WAIS-V; Wechsler, 2024). Finally, in relation to the differential diagnosis between MCI and ADHD, there is no test created and validated for that purpose. This makes it difficult to deal with these older patients without a previously established diagnosis. A potential approach with them would be to compile (through them and potential proxies) a life history and the main difficulties encountered (in the search for possible symptoms of ADHD vs MCI), as well as a selection of relevant neuropsychological tests focused on distinguishing between the predominant cognitive processes in MCI (such as memory) and ADHD (such as attention and inhibition). All these efforts will hopefully lead to a better understanding of the condition that will make it possible for an early diagnosis and a better-tailored treatment plan for older adults with ADHD.

In summary, this systematic review aimed to characterize ADHD in older adults, putting emphasis on deficits in specific individual cognitive domains. While deficits in attention, episodic and working memory seem to be more established in this condition, a characterization of clear deficits in most executive functions is still controversial. Further research with clearly agreed common neuropsychological assessment protocols is needed to characterize ADHD in older adults versus other age-associated conditions like neurodegenerative diseases, as well as to clearly determine the frontiers for significant cognitive changes between younger and older adults with ADHD. The implications of this research, we believe, should lead future research to accurately identify clinical cases of ADHD in older adults and establish targeted treatment protocols that consider potential clinical confounders (comorbidities or other age-associated cognitive disorders) as well as other health risks and side effects. Ultimately, getting a clear characterization of the disorder in older adults will allow the establishment of clearer clinical guidelines and more targeted pharmacological and non-pharmacological treatment strategies.
